# The cooperative promotion induced by Na modification of the Pd–Ce/USY catalyst for HCHO oxidation at room temperature

**DOI:** 10.1039/d6ra03944h

**Published:** 2026-08-03

**Authors:** Longbao Jiang, Jiani Zheng, Qiyong Li, Jia Liu, Fuda Li, Jiangfei Deng, Huangjian Luo, Wangchuan Xiao, Xiaofeng Liu

**Affiliations:** a Fujian Provincial Key Laboratory of Resources and Environmental Monitoring and Sustainable Management and Utilization, Sanming University Sanming Fujian 365004 China xiaofengliu@fjsmu.edu.cn; b School of Resources & Chemical Engineering, Sanming University Sanming 365004 China xwc@fjsmu.edu.cn; c Cleaner Production Technology Engineering Research Center of Fujian Universities Sanming 365004 China; d Fujian Engineering Research Center of Fluorine-containing Advanced Materials, Sanming University Sanming 365004 China; e Center for Excellence in Regional Atmospheric Environment, Key Laboratory of Urban Pollutant Conversion, Institute of Urban Environment, Chinese Academy of Sciences Xiamen 361021 China

## Abstract

Room-temperature catalytic oxidation is an important strategy for the efficient removal of indoor low-concentration HCHO. However, catalysts often suffer from insufficient generation of reactive oxygen species, accumulation of reaction intermediates, and decreased stability at low temperatures. In this study, Na modified Pd–Ce/USY catalysts were constructed using USY zeolite as the support, and the cooperative effect of Na and Ce on room-temperature HCHO oxidation was systematically investigated. Activity evaluation showed that the introduction of an appropriate amount of Na significantly enhanced the HCHO oxidation performance of the catalyst, and the Pd–Na–Ce/USY-R catalyst maintained 100% HCHO conversion during a 12 h continuous reaction. Various characterization results revealed that Na modification changes the framework acid–base properties and the interfacial oxygen environment, increases the population of defect-related oxygen species, and improves the low-temperature conversion of adsorbed HCHO-derived intermediates. The enhanced performance is attributed to the cooperative functions of Pd sites for HCHO/O_2_ activation, CeO_*x*_-associated sites for interfacial oxygen transfer, and Na-modified surface sites for accelerating intermediate removal.

## Abstract 2

Room-temperature catalytic oxidation is an important strategy for the efficient removal of indoor low-concentration HCHO.

## Graphical abstract

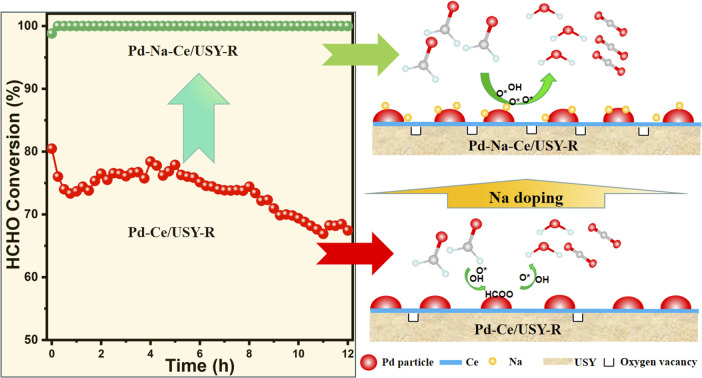

## 1 Introduction

Formaldehyde (HCHO) is a typical indoor volatile organic pollutant that is widely present in residential, office, and public buildings.^1^ Its main sources include wood-based panels, adhesives, paints, wallpaper, flooring, furniture, and textile decorative materials.^2^ As modern buildings have become more airtight, indoor air exchange has decreased. As a result, HCHO remaining in or slowly released from decoration materials can easily accumulate indoors over a long period, leading to persistent pollution.^3^ Even long after renovation has been completed, it may continue to be released from the interior of panels and adhesives.^4^ HCHO is highly chemically active and biologically toxic, and long-term exposure to low concentrations of HCHO may have adverse effects on human health.^5^ It can irritate the eyes, nose, throat, and respiratory mucosa, causing symptoms such as tearing, coughing, chest tightness, and allergic reactions. Long-term exposure may also affect the immune and nervous systems and increase the risk of chronic respiratory diseases.^1^ For elderly people, children, pregnant women, and individuals with respiratory diseases, the health risks associated with long-term exposure to low concentrations of HCHO are even more significant.^6^ Therefore, developing HCHO purification technologies that are efficient, stable, energy-saving, and suitable for room-temperature conditions is of great importance for improving indoor air quality and protecting human health.^7^

At present, the common methods for removing indoor HCHO pollution mainly include adsorption, photocatalysis, plasma treatment, and catalytic oxidation.^7^ Adsorption is simple to operate and relatively low in cost, and it can reduce HCHO concentration within a short time. However, it essentially relies on the physical enrichment of pollutants. Once the adsorbent becomes saturated, secondary release may occur, so regular regeneration or replacement is required.^8^ Photocatalytic technology uses photogenerated electrons and holes to produce highly oxidizing radicals, which gradually oxidize and decompose HCHO. However, its practical application is limited by light utilization efficiency, catalyst stability, and mass transfer efficiency in complex indoor environments.^9^ Plasma technology has a strong ability to activate pollutants, but it may also lead to problems such as ozone formation, by-products, and relatively high energy consumption.^10^ By contrast, catalytic oxidation can deeply convert HCHO into CO_2_ and H_2_O, offering advantages such as complete removal, low secondary pollution, and high reaction selectivity.^11^

Among catalytic oxidation technologies, room-temperature catalytic oxidation has attracted particular attention.^12^ This technology can achieve deep HCHO purification under ambient conditions without additional heating, meeting the requirements of indoor air purification devices for low energy consumption, safety, and continuous operation. However, HCHO molecules are difficult to activate at room temperature, and the reaction process usually involves multiple steps, including HCHO adsorption, C–H bond activation, oxygen activation, surface intermediate transformation, and final CO_2_ formation.^13^ If the catalyst cannot provide sufficient active oxygen species in time, HCHO may readily form surface intermediates such as dioxymethylene (DOM), formate, carbonate, or bicarbonate species.^14,15^ Therefore, improving O_2_ activation ability, enhancing surface oxygen migration, promoting deep oxidation of intermediates, and suppressing the accumulation of intermediates species are key issues in the study of HCHO catalytic oxidation.^16^

Noble metal catalysts show strong potential for room-temperature catalytic oxidation of HCHO because of their excellent low-temperature oxidation ability.^12^ Noble metals such as Pt, Pd, Au, and Ag can all serve as active components for HCHO oxidation. Among them, Pt-based catalysts usually show high low-temperature activity, but their cost is relatively high. Au-based catalysts are highly sensitive to particle size and metal-support interactions. Pd-based catalysts combine good O_2_ activation ability, C–H bond activation ability, and a relatively lower noble metal cost, making them important candidate systems for constructing room-temperature HCHO oxidation catalysts.^17^ However, single Pd active sites may still face problems during long-term reaction, including insufficient oxygen species supply, limited ability to remove intermediates, and aggregation of metal particles.^18^ To further improve the activity and stability of Pd-based catalysts, coupling Pd with metal oxides that have oxygen storage-release capacity and reversible redox properties is an important strategy for enhancing their room-temperature oxidation performance.^19^

CeO_2_ is a typical rare-earth oxide with a reversible Ce^4+^/Ce^3+^ redox cycle, strong oxygen storage and release capacity, and abundant oxygen vacancy structures. It is commonly used as a promoter or support component in low-temperature oxidation reactions.^20^ CeO_*x*_ can adsorb and activate O_2_ through oxygen vacancies to generate surface active oxygen species. At the same time, its lattice oxygen can also participate in oxidation reactions and be replenished by gas-phase O_2_. The interfacial structure formed between Pd and CeO_*x*_ helps promote electron transfer and oxygen migration, thereby improving the low-temperature oxidation efficiency of HCHO.^19^ In particular, Pd sites can promote the adsorption and initial activation of HCHO molecules, while CeO_*x*_ can provide mobile oxygen species and accelerate the further conversion of intermediates such as formate into CO_2_.^21^ However, the oxygen vacancy concentration, surface acid–base properties, and interfacial oxygen migration ability of CeO_*x*_ are still affected by the preparation method, support structure, and promoter regulation. Therefore, further modification is needed to enhance the reaction efficiency of the Pd–CeO_*x*_ interface.^22^

Alkali metal modification is an effective way to tune the surface properties of catalysts.^23^ The introduction of Na may change the local electronic structure of the catalyst through ion exchange, surface deposition, or interaction with the support framework, thereby regulating the surface acid–base properties and the distribution of oxygen species.^24^ On the one hand, an appropriate amount of Na can enhance surface basic oxygen sites, which is favorable for the adsorption and activation of polar HCHO molecules. On the other hand, Na may also affect the electronic interaction between Pd and CeO_*x*_, promote the formation of oxygen vacancies and interfacial oxygen migration, and thus improve low-temperature oxidation activity.^25^ However, the role of Na is strongly dependent on its content.^26^ When the Na content is too low, its regulatory effect on surface acid–base properties and oxygen species is limited. When the Na content is too high, it may cover the active sites of Pd or CeO_*x*_, or even block the pores of the support, thereby hindering the diffusion of HCHO, O_2_, and reaction intermediates.^27^ Therefore, optimizing the Na content and clarifying its synergistic interaction with Pd and Ce are of great significance for designing efficient and stable catalysts for room-temperature HCHO oxidation.

In addition to active components and promoters, the support structure also has an important influence on catalytic performance.^28^ USY zeolite has a high specific surface area, good thermal stability, and adjustable acid sites. Its microporous structure is beneficial for the dispersion of active components and the adsorption of gas molecules.^29^ Meanwhile, a combined microporous–mesoporous structure formed after appropriate treatment can improve molecular diffusion and mass transfer, reducing the retention of reaction intermediates within the pores.^30^ For HCHO oxidation, USY zeolite can not only provide dispersion sites for Pd, Na, and Ce species, but also offer a suitable pore environment for the contact, adsorption, and conversion of HCHO and O_2_. Therefore, constructing a Pd–Na–Ce multicomponent catalytic system using USY as the support is expected to combine the synergistic advantages of noble metal activation, CeO_*x*_-assisted oxygen migration, and Na-regulated surface chemistry.

In our previous study, Ce modification was demonstrated to independently enhance the activity and stability of Pd/USY for room-temperature HCHO oxidation.^19^ The present work further investigates whether Na modification can improve the interfacial oxygen environment and intermediate-conversion ability of this previously established Pd–Ce/USY catalyst. Therefore, in this study, USY zeolite was used as the support to construct Na modified Pd–Ce/USY catalysts. Characterization techniques including N_2_ adsorption–desorption, HAADF-STEM, XRD, H_2_-TPR, O_2_-TPD, ESR, XPS, and HCHO-TPD were employed to systematically analyze the relationships among the pore structure, crystalline phase composition, elemental distribution, oxygen vacancy concentration, surface oxygen species, and transformation behavior of reaction intermediates. This study aims to clarify the mechanism by which Na–Ce cooperative affects the construction of Pd active sites and improves low-temperature deep oxidation performance, providing experimental evidence and theoretical guidance for the rational design of efficient room-temperature HCHO oxidation catalysts.

## 2 Experimental section

### 2.1 Catalyst preparation and characterization

Details of the preparation and characterization of the catalysts are described in the SI.

### 2.2 Activity test

The experimental conditions for the activity testing of the catalysts for HCHO catalytic oxidation were according to our previous work.^18^

## 3 Results and discussion

### 3.1 HCHO oxidation testing

**Fig. 1:**
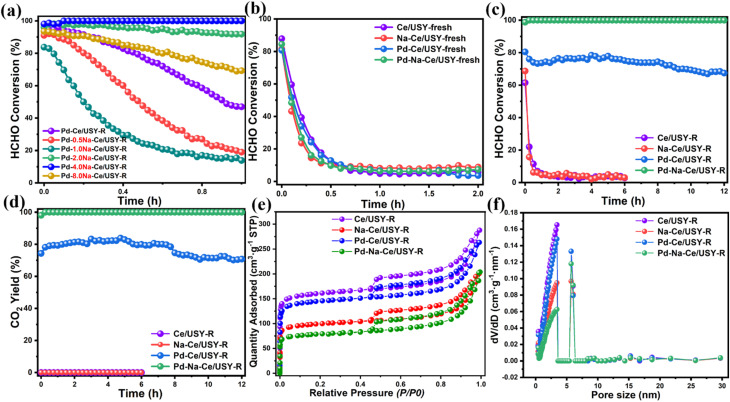
(a) HCHO conversion on Pd–Ce/USY-R, Pd–0.5Na/USY-R, Pd–1.0Na/USY-R, Pd–2.0Na/USY-R, Pd–4.0Na/USY-R, Pd–8.0Na/USY-R catalysts. Reaction conditions: 180 ppm HCHO, 20% O_2_, 35% RH, He balance, WHSV of 600 000 mL g^−1^ h^−1^, 25 °C. (b) HCHO conversion on Ce/USY-fresh, Na–Ce/USY-fresh, Pd–Ce/USY-fresh and Pd–Na–Ce/USY-fresh catalysts. Reaction conditions: 150 ppm HCHO, 20% O_2_, 35% RH, He balance, WHSV of 300 000 mL g^−1^ h^−1^, 25 °C. (c) HCHO conversion and (d) CO_2_ yield on Ce/USY-R, Na–Ce/USY-R, Pd–Ce/USY-R and Pd–Na–Ce/USY-R catalysts. Reaction conditions: 150 ppm HCHO, 20% O_2_, 35% RH, He balance, WHSV of 300 000 mL g^−1^ h^−1^, 25 °C. (e) and (f) Nitrogen adsorption–desorption isotherms and pore size distributions of Ce/USY-R, Na–Ce/USY-R, Pd–Ce/USY-R and Pd–Na–Ce/USY-R catalysts.

Fig. 1a shows the effect of different Na contents on the room-temperature HCHO oxidation performance of the Pd–Na–Ce/USY-R catalysts. As shown in the Fig. 1a, the introduction of Na significantly regulates the activity and stability of the catalysts, but the catalytic performance does not increase monotonically with increasing Na content. The Na-free Pd–Ce/USY-R catalyst shows a high HCHO conversion at the initial stage of the reaction, but the conversion decreases markedly with time and finally drops to below approximately 50%. After the introduction of a small amount of Na, the catalytic performance is not effectively improved. Both Pd–0.5Na–Ce/USY-R and Pd–1.0Na–Ce/USY-R exhibit low activity and obvious deactivation, with Pd–1.0Na–Ce/USY-R maintaining only about 10–20% conversion at the later stage of the test. When the Na content increases to an appropriate level, the catalytic performance improves significantly. Pd–2.0Na–Ce/USY-R shows an initial HCHO conversion close to 100%, which gradually decreases with reaction time and finally stabilizes at approximately 90%. In contrast, Pd–4.0Na–Ce/USY-R exhibits the best activity and stability, with HCHO conversion remaining close to 100% throughout the test. When the Na content is further increased to 8.0 wt%, the HCHO conversion of Pd–8.0Na–Ce/USY-R slowly decreases from approximately 90% to about 70%, and its activity is lower than that of Pd–4.0Na–Ce/USY-R. The catalytic performance follows the order: Pd–4.0Na–Ce/USY-R > Pd–2.0Na–Ce/USY-R > Pd–8.0Na–Ce/USY-R > Pd–Ce/USY-R > Pd–0.5Na–Ce/USY-R > Pd–1.0Na–Ce/USY-R. These results indicate that the role of Na is not a simple alkali-metal doping effect, but depends on the formation of a suitable synergistic structure among Na, CeO_*x*_, and Pd. An appropriate amount of Na helps optimize the Pd–CeO_*x*_ interfacial environment, promoting the generation of active oxygen species and the conversion of reaction intermediates. In contrast, either insufficient or excessive Na weakens this synergistic effect. For clarity in the following discussion, the optimized Pd–4.0Na–Ce/USY-R catalyst was hereafter denoted as Pd–Na–Ce/USY-R.

To clarify the effect of reduction treatment on the formation of catalytic active sites, Fig. 1b compares the HCHO oxidation performance of different unreduced samples. Ce/USY-fresh, Na–Ce/USY-fresh, Pd–Ce/USY-fresh, and Pd–Na–Ce/USY-fresh all show relatively high apparent HCHO conversion at the initial stage of the reaction, approximately 80–90%. However, as the reaction proceeds, the conversion of all samples decreases rapidly and drops to about 10–25% within 0.3–0.5 h. After 0.6 h of reaction, the four unreduced samples stabilize at low conversion levels of only about 4–10%. This behavior indicates that the high initial conversion over the unreduced samples may be associated with surface adsorption or the consumption of a small amount of initially available active oxygen, rather than a stable catalytic oxidation process. As the available oxygen species are depleted or surface intermediates accumulate, the catalysts deactivate rapidly. The small differences in the final conversion among the unreduced samples suggest that, in the absence of reduction activation, the introduction of Pd, Na, or Ce alone is insufficient to construct highly efficient active sites for room-temperature HCHO oxidation.

Fig. 1c further compares the HCHO oxidation activity of different catalysts after reduction treatment, and Fig. 1d shows the corresponding CO_2_ yield rates. Ce/USY-R and Na–Ce/USY-R both exhibit obvious deactivation. Their initial HCHO conversions are approximately 60% and 70%, respectively, but rapidly decrease to below 10% within a short time and then remain at a low level of about 2–6% for the rest of the test. This result indicates that the Ce or Na–Ce system alone is unable to achieve sustained deep oxidation of HCHO at room temperature. After the introduction of Pd, the catalytic performance is significantly improved. Pd–Ce/USY-R shows an initial HCHO conversion of approximately 80%, which then remains mainly in the range of 70–78%. After 12 h, the conversion is still maintained at about 67–70%, indicating that Pd is the key active component for room-temperature HCHO oxidation. With the further introduction of Na, Pd–Na–Ce/USY-R exhibits the highest and most stable conversion. Its initial HCHO conversion is close to 98%, then rapidly reaches nearly 100% and remains at this level for a long time, with almost no obvious decline within 12 h. As shown in Fig. 1d, the HCHO conversion and CO_2_ formation rate of each sample show a clear correspondence, indicating that the converted HCHO is fully oxidized to CO_2_. Therefore, the HCHO removal performance follows the order: Pd–Na–Ce/USY-R > Pd–Ce/USY-R ≫ Na–Ce/USY-R ≈ Ce/USY-R. These results demonstrate that the presence of Pd determines the basic activity for room-temperature HCHO oxidation, while the Na modification Pd–Ce/USY modification further improves the stability and deep oxidation ability of the Pd-based catalyst.

### 3.2 Physicochemical properties

Fig. 1e shows the N_2_ adsorption–desorption isotherms of the catalysts. All four samples exhibit a sharp uptake in the low relative pressure region of *P*/*P*_0_ < 0.05, indicating that the typical microporous structure of the USY zeolite is retained. In the medium- and high-relative-pressure regions, the adsorption capacity continues to increase gradually and rises further at *P*/*P*_0_ > 0.8, accompanied by a clear adsorption–desorption hysteresis loop. This suggests the presence of mesopores in the samples. Therefore, these samples display combined type I/IV adsorption isotherm characteristics with an H4-type hysteresis loop, indicating a hierarchical micro–mesoporous structure. The pore size distribution results in Fig. 1f show that the pore sizes of the four samples are mainly concentrated in the range of 0–10 nm, confirming that they are dominated by micro–mesoporous structures. According to the specific surface area data in Table 1, the order is as follows: Pd–Ce/USY-R (572.6 m^2^ g^−1^) > Ce/USY-R (548.9 m^2^ g^−1^) > Na–Ce/USY-R (332.3 m^2^ g^−1^) > Pd–Na–Ce/USY-R (297.0 m^2^ g^−1^). The substantial decrease in *S*_BET_ after Na-containing modification is likely associated with ion exchange and the deposition of Na-, Ce-, and Pd-containing species on the internal pore surfaces or near pore entrances, which partially occupy or block the micropores. However, the characteristic low-pressure uptake and the type-I/type-IV isotherms with H4-type hysteresis are retained, indicating that the hierarchical micro–mesoporous framework is not completely obstructed. Therefore, the decrease in *S*_BET_ surface area represents a reduction in the N_2_-accessible surface area rather than complete loss of molecular transport pathways. Although Pd–Na–Ce/USY-R possesses the lowest *S*_BET_, it exhibits the highest HCHO conversion and CO_2_ yield, demonstrating that the catalytic activity is not governed by the total surface area. Instead, the quality and accessibility of Pd–CeO_*x*_ interfacial sites, together with defect-related oxygen species and enhanced intermediate oxidation, play more important roles.

**Table 1:** Specific surface area (*S*_BET_) of four samples

Catalysts	*S*_BET_ (m^2^ g^−1^)
Ce/USY-R	548.9
Na–Ce/USY-R	332.3
Pd–Ce/USY-R	572.6
Pd–Na–Ce/USY-R	297.0

**Fig. 2:**
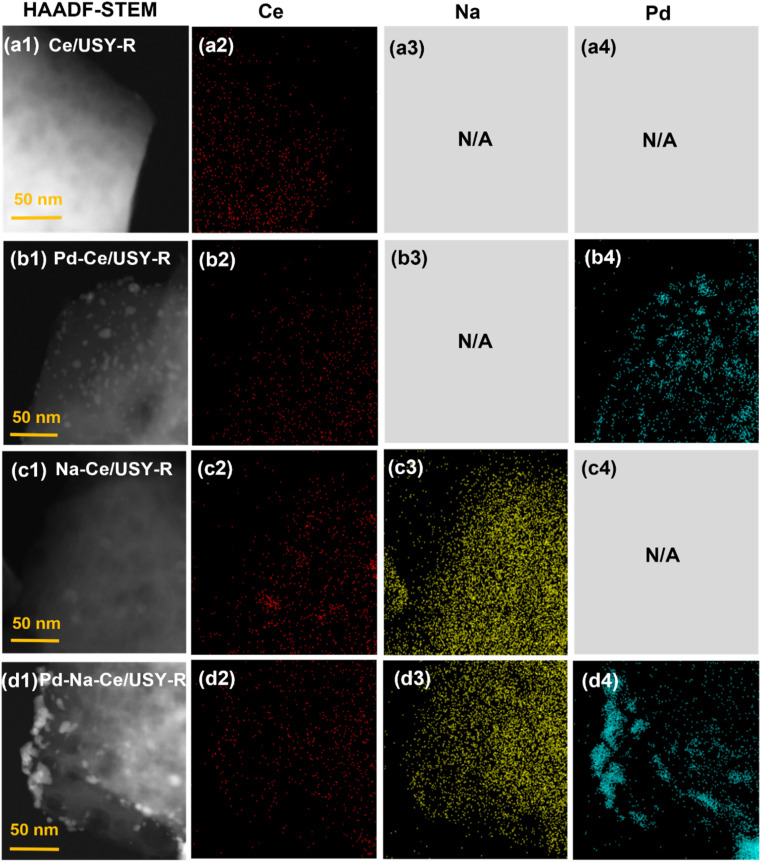
HAADF-STEM images and EDS elemental mapping of catalysts. Row (a) shows the maps for Ce/USY-R; row (b) for Pd–Ce/USY-R; row (c) for Na–Ce/USY-R; and row (d) for Pd–Na–Ce/USY-R. Columns display EDS elemental mapping, Ce (red), Na (yellow), and Pd (blue) maps, respectively. Grey squares indicate data not acquired (N/A).

Fig. 2 shows the HAADF-STEM images and EDS elemental mapping results of the samples. For all four samples, the Ce signal covers the entire particle region, and no obvious aggregation of large particles is observed, indicating that the Ce species are well dispersed on the support. Similarly, Na is also well dispersed in the Na–Ce/USY-R and Pd–Na–Ce/USY-R samples. According to the distribution of Pd, Pd–Ce/USY-R shows better Pd dispersion, whereas the Pd distribution in Pd–Na–Ce/USY-R is relatively less uniform. Overall, the distribution regions of Pd, Na, Ce, and the USY framework elements largely overlap, and no large-scale separation of independent phases or severe aggregation is observed. This well-dispersed state is favorable for the formation of Pd–CeO_*x*_ interfaces and provides a structural basis for Na to regulate the interfacial electronic structure and oxygen migration behavior.

**Fig. 3:**
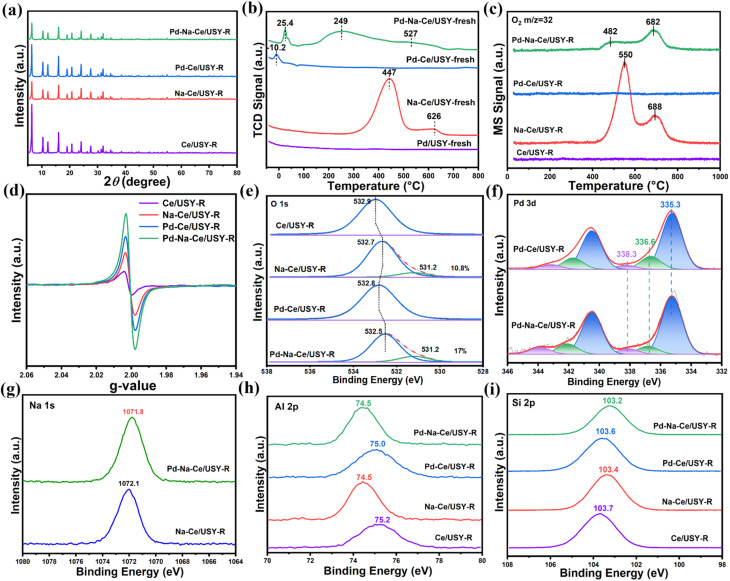
(a) XRD patterns, (b) H_2_-TPR profiles, (c) O_2_-TPD profiles, (d) EPR profiles of Ce/USY-R, Na–Ce/USY-R, Pd–Ce/USY-R and Pd–Na–Ce/USY-R catalysts. (e)–(i) XPS spectra of O 1s, Pd 3d, Na 1s, Al 2p, Si 2p of different catalysts.

Fig. 3a shows the XRD patterns of Ce/USY-R, Na–Ce/USY-R, Pd–Ce/USY-R, and Pd–Na–Ce/USY-R. All four samples retain the main diffraction peaks of the USY zeolite, with relatively well-defined peak shapes, indicating that the main crystalline framework of USY is not significantly damaged after modification. Compared with Ce/USY-R, some diffraction peak intensities change after the introduction of Na and Pd, suggesting that metal modification has a certain effect on the crystallinity or local structure of the samples, but does not lead to collapse of the main framework. No distinguishable diffraction peaks attributable to crystalline Pd or PdO are observed for Pd–Ce/USY-R or Pd–Na–Ce/USY-R. However, the absence of detectable XRD reflections should not be interpreted as direct evidence for uniformly dispersed Pd. The HAADF-STEM and EDS results (Fig. 2) show that Pd–Ce/USY-R has a relatively uniform Pd distribution, whereas local Pd-rich regions or aggregates are present in Pd–Na–Ce/USY-R. The apparent dimensions of these high-contrast regions represent projected agglomerates and do not necessarily correspond to the coherent crystallite sizes detected by XRD. The lack of Pd/PdO diffraction peaks may therefore result from the low Pd content, poor crystallinity, peak broadening, and/or the presence of smaller crystalline subdomains within the observed aggregates.

Fig. 3b shows the H_2_-TPR profiles of different unreduced samples. Ce/USY-fresh shows no obvious reduction peak within the tested temperature range, indicating that it contains few low-temperature reducible species. Na–Ce/USY-fresh mainly exhibits reduction peaks in the medium- and high-temperature regions, with a peak at approximately 447 °C and a weak reduction signal at around 626 °C. This indicates that the reducible species in the Na–Ce system are mainly reduced at relatively high temperatures and are therefore difficult to provide sufficient active oxygen for the room-temperature reaction. The introduction of Pd significantly improves the low-temperature reducibility of the samples. Pd–Ce/USY-fresh shows a distinct low-temperature reduction peak at approximately 10.2 °C, suggesting that Pd species can effectively promote H_2_ activation and the reduction of surface oxygen species. Pd–Na–Ce/USY-fresh exhibits more complex reduction behavior, with several reduction peaks at approximately 25.4, 249, and 527 °C. This indicates that the coexistence of Pd and Na–Ce leads to the formation of different types of reducible oxygen species or metal–oxygen interfacial structures.^26^ In particular, the presence of low- and medium-temperature reduction peaks suggests that this sample has stronger oxygen mobility and low-temperature redox ability. Combined with the catalytic results, Pd–Ce/USY-R and Pd–Na–Ce/USY-R, both of which show clear low-temperature reduction features, exhibit much higher HCHO conversion than the Pd-free samples. Among them, Pd–Na–Ce/USY-R shows the highest activity, indicating that low-temperature reducibility is closely related to room-temperature HCHO oxidation performance. The Na modification Pd–Ce/USY not only changes the reduction behavior of oxygen species around Pd, but may also promote oxygen cycling at the Pd–CeO_*x*_ interface, thereby enhancing the deep oxidation of HCHO.^21^

Fig. 3c shows the O_2_-TPD profiles of different samples. Ce/USY-R and Pd–Ce/USY-R show no obvious O_2_ desorption peaks within the tested temperature range, indicating that their detectable oxygen desorption signals are weak. Na–Ce/USY-R exhibits a clear medium-to high-temperature O_2_ desorption peak, with the main peak located at approximately 550 °C, together with a weaker high-temperature desorption peak at around 688 °C. This suggests that the introduction of Na can enhance the adsorption or stabilization of certain oxygen species. Pd–Na–Ce/USY-R displays more distinct multi-peak O_2_ desorption features, with desorption peaks at approximately 482 and 682 °C. Notably, the peak at 482 °C shifts markedly to a lower temperature compared with the 550 °C peak of Na–Ce/USY-R, indicating that the coexistence of Pd and Na–Ce improves the mobility and release ability of oxygen species.^31^ It should be noted that although Na–Ce/USY-R shows clear O_2_ desorption peaks, its room-temperature HCHO oxidation activity remains low. This indicates that oxygen desorption ability alone does not determine the catalytic performance.^15^ A significant improvement in room-temperature HCHO oxidation efficiency can only be achieved when Pd active sites are effectively coupled with oxygen species regulated by Na–Ce.^32^

The ESR spectra in Fig. 3d reveal the differences in defect structures among the samples. All four samples show a signal near *g* ≈ 2.00, indicating the presence of defect sites associated with oxygen vacancies or unpaired electrons. The signal intensity follows the order: Pd–Na–Ce/USY-R > Pd–Ce/USY-R > Na–Ce/USY-R > Ce/USY-R. This trend is generally consistent with the order of HCHO oxidation activity, suggesting that an increased number of oxygen vacancies or defect sites is beneficial for O_2_ adsorption and activation, as well as the formation of active oxygen species. Among the samples, Pd–Na–Ce/USY-R exhibits the strongest ESR signal and the highest HCHO conversion, indicating that the Na–Ce cooperative effect can enhance the defect-related oxygen structure in the Pd-based catalyst, thereby promoting the room-temperature oxidation reaction.

Fig. 3e shows the O 1s XPS spectra of the different samples. The main O 1s peaks of the four samples are located at approximately 532.5–532.9 eV, which can be assigned to oxygen species such as surface oxygen on the support, adsorbed oxygen, or hydroxyl oxygen.^33^ Compared with Ce/USY-R, the main O 1s peak of Pd–Na–Ce/USY-R shifts from 532.9 to 532.5 eV, showing the most pronounced shift toward lower binding energy. This indicates that the co-introduction of Na and Pd changes the electronic environment of surface oxygen species.^34^ The peak deconvolution results show that Na–Ce/USY-R and Pd–Na–Ce/USY-R exhibit a low-binding-energy oxygen species peak at around 531.2 eV, with relative contents of 10.8% and 17.0%, respectively. The markedly higher proportion of this oxygen species in Pd–Na–Ce/USY-R suggests that the co-modification with Pd and Na–Ce favors the formation of more defect-related oxygen species or highly active surface oxygen species. Combined with its highest HCHO conversion, the increase in low-binding-energy oxygen species may promote the low-temperature oxidation of formaldehyde-derived intermediates and improve CO_2_ formation efficiency.^7^

Further analysis of the Pd 3d, Na 1s, Si 2p, and Al 2p XPS spectra. As shown in Fig. 3f, that both Pd–Ce/USY-R and Pd–Na–Ce/USY-R exhibit clear Pd 3d characteristic peaks, confirming the successful loading of Pd species on the catalyst surface. The Pd 3d peak positions of the two samples are nearly identical, while their peak intensities differ, suggesting that the introduction of Na may affect the surface exposure state or local electronic environment of Pd.^35^ In the Na 1s spectra (Fig. 3g), Na–Ce/USY-R and Pd–Na–Ce/USY-R show Na 1s signals at 1072.1 and 1071.8 eV, respectively. After Pd introduction, the Na 1s peak slightly shifts toward lower binding energy, indicating a certain electronic interaction between Pd and Na.^36^ In the Al 2p and Si 2p spectra (Fig. 3h and i), Na–Ce/USY-R and Pd–Na–Ce/USY-R both show varying degrees of shifts toward lower binding energy compared with Ce/USY-R. This indicates that Na may change the electronic environment of Si and Al near the USY framework through ion exchange or surface modification, and further affect the dispersion of metal species and the interfacial structure. Overall, the XPS results demonstrate clear interactions among Pd, Na, Ce, and the USY support in Pd–Na–Ce/USY-R. The introduction of Na not only regulates the surface electronic structure of the support, but also increases the proportion of low-binding-energy oxygen species.^26^ This optimization of the electronic structure and oxygen species composition is an important reason for the significantly enhanced room-temperature HCHO oxidation performance.^32^

### 3.3 Effects of H_2_O and O_2_ on HCHO oxidation

**Fig. 4:**
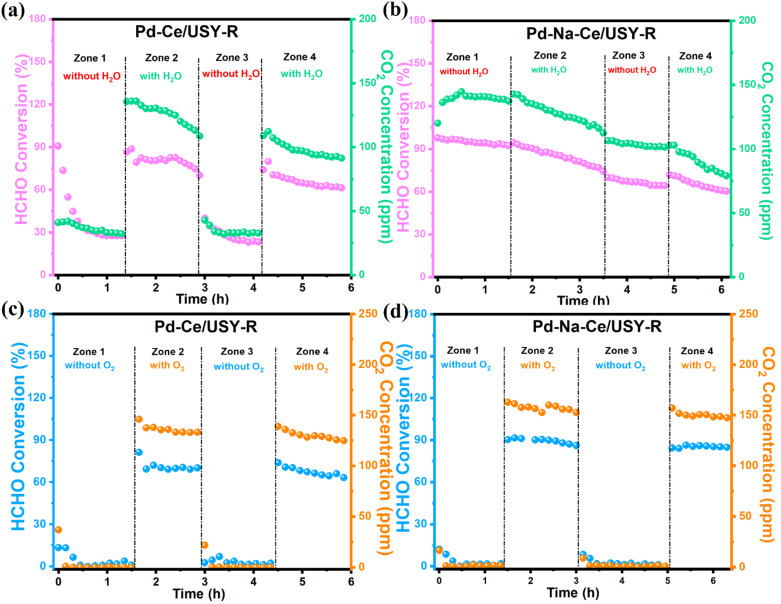
(a) and (b) Effect of H_2_O on the HCHO oxidation activity of Pd–Ce/USY-R and Pd–Na–Ce/USY-R, respectively. Reaction conditions: 180 ppm HCHO, 20% O_2_, 0 or 35% RH, He balance, WHSV of 400 000 mL g^−1^ h^−1^, 25 °C; (c) and (d) effect of O_2_ on the HCHO oxidation activity of Pd–Ce/USY-R and Pd–Na–Ce/USY-R. Reaction conditions: 180 ppm HCHO, 35% RH, 0 or 20% O_2_, He balance, WHSV of 400 000 mL g^−1^ h^−1^, 25 °C.

Fig. 4a and b investigate the effect of water vapor on room-temperature HCHO oxidation over Pd–Ce/USY-R and Pd–Na–Ce/USY-R through H_2_O on/off cycling experiments. For Pd–Ce/USY-R, under H_2_O-free conditions, the HCHO conversion rapidly decreases from a relatively high initial value and stabilizes at approximately 30% in zone 1. After the introduction of H_2_O, the conversion increases markedly to about 80–85% in zone 2. When H_2_O is removed again, the HCHO conversion decreases to approximately 20–25% in zone 3. Upon reintroducing H_2_O, the conversion recovers to about 60–70% in zone 4. These results indicate that H_2_O has a clear promotional effect on Pd–Ce/USY-R, which may be related to the participation of water molecules in the formation of surface hydroxyl groups, the further conversion of intermediates such as formate species, and the acceleration of the surface active oxygen cycle. In contrast, Pd–Na–Ce/USY-R already exhibits high HCHO conversion and CO_2_ formation ability under H_2_O-free conditions. In zones 1 and 3, its HCHO conversions are approximately 90–100% and 60–75%, respectively, which are much higher than those of Pd–Ce/USY-R under dry conditions. After H_2_O is introduced, the catalyst still maintains a high reaction level, but the promotional effect is less pronounced than that observed for Pd–Ce/USY-R. Meanwhile, both the conversion and CO_2_ concentration show a certain decrease as the cycles proceed. These results suggest that, after Na modification Pd–Ce/USY, the catalyst possesses stronger low-temperature oxygen activation and intermediate conversion abilities, thereby reducing its dependence on external H_2_O. At the same time, H_2_O may also compete with HCHO or O_2_ for adsorption on some active sites, leading to certain fluctuations in activity during long-term operation.

The O_2_ on/off cycling experiments shown in Fig. 4c and d further demonstrate the importance of gas-phase O_2_ in room-temperature HCHO oxidation. In the absence of O_2_, the HCHO conversion over Pd–Ce/USY-R rapidly decreases to nearly zero (zone 1). After O_2_ is introduced, the HCHO conversion quickly recovers to approximately 65–70% (zone 2). When O_2_ is switched off again, both the conversion and CO_2_ concentration decrease (zone 3); upon reintroducing O_2_, the catalytic activity is restored (zone 4). Pd–Na–Ce/USY-R shows a similar response to O_2_, but with higher overall HCHO conversion and CO_2_ formation. During the O_2_-containing stage, its HCHO conversion rapidly increases to approximately 85–90%. These results indicate that O_2_ is an essential oxygen source for the sustained room-temperature oxidation of HCHO, and that Pd–Na–Ce/USY-R has a stronger ability to adsorb, activate, and convert O_2_. The Na–Ce cooperative effect may improve the reaction efficiency of the Pd-based catalyst by increasing the concentration of oxygen vacancies, promoting O_2_ activation, and facilitating interfacial oxygen migration.

### 3.4 The oxidation mechanism

**Fig. 5:**
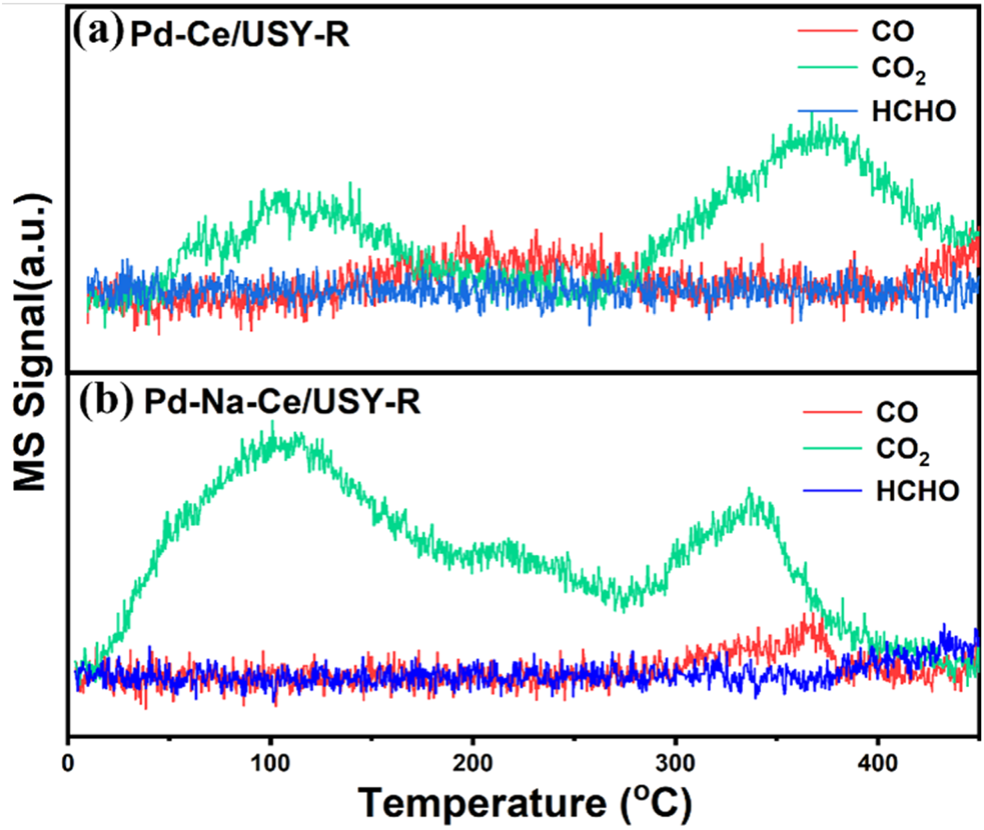
HCHO-TPD profiles of (a) Pd–Ce/USY-R and (b) Pd–Na–Ce/USY-R catalysts.

The HCHO-TPD results (Fig. 5) shown that, after HCHO/O_2_ pretreatment, CO_2_ is the main desorption/reaction product for both catalysts during temperature-programmed heating under a He atmosphere, whereas the HCHO and CO signals are relatively weak. This indicates that the pre-adsorbed HCHO species mainly exist on the catalyst surface as oxygen-containing intermediates, such as DOM, formate, or carbonate species, which are further oxidized or decomposed into CO_2_ during heating.^13^ For Pd–Ce/USY-R, CO_2_ release is mainly concentrated in the medium-to high-temperature range of 300–400 °C, indicating that the carbon-containing oxygenated intermediates formed on its surface are relatively stable and difficult to convert at low temperature; therefore, a higher temperature is required for their further oxidation to CO_2_.^37^ Such stable intermediates can readily cover Pd or CeO_*x*_ active sites during the room-temperature reaction, leading to a decrease in catalytic activity.^15^ The weak CO signal may originate from the incomplete oxidation or thermal decomposition of strongly adsorbed formate or carbonate species.^38^ In contrast, Pd–Na–Ce/USY-R shows a markedly enhanced low-temperature CO_2_ release peak in the range of 50–160 °C, while the medium- and high-temperature CO_2_ peaks are relatively weakened. This suggests that Na modification Pd–Ce/USY significantly promotes the low-temperature activation and deep oxidation of HCHO-derived intermediates.^36^ This catalyst can further convert surface carbon-containing oxygenated species into CO_2_ at lower temperatures, thereby reducing the accumulation of stable intermediates and lowering the likelihood of active-site coverage.^26^ This result is highly consistent with its high activity and excellent stability in room-temperature HCHO oxidation.^19^

Based on the H_2_O/O_2_ switching experiments and HCHO-TPD results, an interfacial reaction pathway is proposed for Pd–Na–Ce/USY-R. HCHO is first adsorbed and activated at or near Pd-containing interfacial sites. Reaction with surface hydroxyl and/or oxygen species produces DOM-like species, which are subsequently converted into surface formate species. Gas-phase O_2_ is activated at Pd and defect-containing Pd–CeO_*x*_ interfacial regions and replenishes reactive interfacial oxygen species required for the continuous oxidation reaction. Na primarily modifies the local acid–base properties of the USY framework and the oxygen environment adjacent to the Pd–CeO_*x*_ interface. CeO_*x*_-associated defect sites are proposed to assist O_2_ adsorption and the transfer or replenishment of interfacial oxygen species. This interpretation is consistent with the enhanced ESR signal, the increased proportion of defect-related surface oxygen species, and the altered H_2_-TPR and O_2_-TPD behavior, although the present *ex situ* measurements do not directly establish a dynamic Ce^4+^/Ce^3+^ redox cycle under reaction conditions. This modification is proposed to facilitate the conversion of strongly adsorbed formate or carbonate species into CO_2_. The markedly enhanced CO_2_ release at 50–160 °C and the weakened high-temperature CO_2_ signal in the HCHO-TPD profile of Pd–Na–Ce/USY-R indicate that HCHO-derived oxygenated intermediates are less stable and can be removed at lower temperature than those on Pd–Ce/USY-R. On Pd–Ce/USY-R, externally supplied H_2_O strongly promotes HCHO conversion, suggesting that water-derived surface hydroxyls assist the transformation of DOM/formate species or the regeneration of active sites. In contrast, Pd–Na–Ce/USY-R retains considerably higher activity under dry-feed conditions. We propose that the Na-modified interfacial environment enables more efficient utilization of intrinsic surface hydroxyls and defect-related oxygen species. In addition, H_2_O generated during HCHO oxidation may replenish surface hydroxyl groups after the reaction is initiated, thereby reducing, but not completely eliminating, the dependence on external H_2_O.

## 4 Conclusions

In summary, the catalytic comparison demonstrates that Pd is essential for sustained room-temperature HCHO oxidation, while an appropriate Na loading markedly enhances the activity and stability of the Pd–Ce/USY-R catalyst. The improvement is associated with changes in the framework acid–base properties, defect-related oxygen species, oxygen mobility, and the low-temperature conversion of adsorbed oxygenated intermediates. The available results support a cooperative interfacial mechanism involving Pd, Na-modified surface sites, and CeO_*x*_-associated oxygen species. This cooperative effect not only improves the low-temperature redox performance of the catalyst, but also promotes the deep oxidation of adsorbed HCHO species at low temperature. As a result, the accumulation of stable intermediates is effectively suppressed, allowing the Pd–Na–Ce/USY-R catalyst to maintain HCHO conversion close to 100% during continuous reaction. Future work may further reveal the electronic interaction mechanism among Na, Ce, and Pd through theoretical calculations and clarify the specific role of each species in the cooperative effect. Such studies will provide important theoretical and experimental guidance for the design of efficient and stable catalysts for room-temperature formaldehyde purification.

## Author contributions

Longbao Jiang: investigation, writing – original draft. Jiani Zheng: software, visualization, writing. Qiyong Li: writing – review and editing. Jia Liu: investigation, software. Fuda Li: investigation. Jiangfei Deng: conceptualization. Huangjian Luo: writing – review and editing. Wangchuan Xiao: supervision, funding acquisition. Xiaofeng Liu: supervision, review & editing, funding acquisition.

## Conflicts of interest

There are no conflicts to declare.

## Supplementary Material

RA-OLF-D6RA03944H-s001

## Data Availability

All data supporting this study are contained within the main text of the article. Supplementary information (SI): details of the preparation and characterization of the catalysts. See DOI: https://doi.org/10.1039/d6ra03944h.
