# Supplementary material for: The cooperative promotion induced by Na modification of the Pd–Ce/USY catalyst for HCHO oxidation at room temperature
Source: RSC Adv. 2026 Aug 3. Online ahead of print. doi: 10.1039/d6ra03944h (PMC13430518; doi:10.1039/d6ra03944h)
Supplement: RA-OLF-D6RA03944H-s001 [file RA-OLF-D6RA03944H-s001.pdf]

Supplementary Information (SI) for RSC Advances.
This journal is © The Royal Society of Chemistry 2026

Supplementary Information

The cooperative promotion induced by Na modification of the Pd－Ce/USY catalyst for HCHO oxidation at room temperature

Longbao Jiang[ab], Jiani Zheng[ab], Qiyong Li[bc], Jia Liu[ab], Fuda Li[ab], Jiangfei Deng[ab], Huangjian Luo[b], Wangchuan Xiao[*bd] and Xiaofeng Liu[*abce]

[a] Fujian Provincial Key Laboratory of Resources and Environmental Monitoring and Sustainable Management and Utilization, Sanming University, Sanming 365004, China

[b] School of Resources & Chemical Engineering, Sanming University, Sanming 365004, China

[c] Cleaner Production Technology Engineering Research Center of Fujian Universities, Sanming 365004, China

[d] Fujian Engineering Research Center of Fluorine-containing Advanced Materials, Sanming University, Sanming 365004, China

[e] Center for Excellence in Regional Atmospheric Environment, Key Laboratory of Urban Pollutant Conversion, Institute of Urban Environment, Chinese Academy of Sciences, Xiamen 361021, China

*Corresponding authors.

Emails: xwc@fjsmu.edu.cn (W. Xiao); xiaofengliu@fjsmu.edu.cn (X. Liu).

Text S1. Catalyst preparation.

The USY zeolites (H type, Si/Al at 11, Nankai University Catalyst Co., Ltd, China) were prepared with hydrochloric acid (HCl AR grade, Sinopharm Chemical Reagent Beijing Co., Ltd, China), cerium nitrate ($Ce(NO_3)_3·6H_2O$, AR grade, Sinopharm Chemical Reagent Beijing Co., Ltd, China) and sodium nitrate ($NaNO_3$, AR grade, Sinopharm Chemical Reagent Beijing Co., Ltd, China).

In a typical procedure, firstly, USY zeolite was dispersed into HCl solution (0.20 M) and constantly stirred in a beaker at 30°C for 6 h. The mass ratio of solid to liquid was fixed at 1:20. After acid treatment, the mixed liquid was filtered and washed with deionized water until neutral. The resultant filter cake was then dried at 110°C overnight in an oven. Secondly, the USY zeolites were impregnated in a cerium nitrate solution (0.5Ce wt.%). After ultrasonication for 30 min and then impregnation under stirring for 4 h, the excess water was removed in a rotary evaporator at 60°C. Then, the sample was dried at 110°C and calcined at 400°C for 4 h. The sample was denoted as Ce/USY. Thirdly, the Ce/USY samples were impregnated in a sodium nitrate solution (4.0Na wt.%). After ultrasonication for 30 min and then impregnation under stirring for 4 h, the excess water was removed in a rotary evaporator at 60°C. Then, the sample was dried at 110°C and calcined at 400°C for 4 h. The sample was denoted as Na-Ce/USY.

The Pd-Ce/USY and Pd-Na-Ce/USY catalysts were prepared by impregnation of Ce/USY and Na-Ce/USY and with $Pd(NO_3)_2·2H_2O$ (Sigma Aldrich) solution, respectively. After ultrasonication for 30 min and then impregnation under stirring for 4 h, the excess water was removed in a rotary evaporator at 60°C and the solid was dried at 110°C overnight. Then, the sample was dried at 110°C and calcined at 400°C for 4 h. Before the catalytic activity testing, the samples were reduced at 350°C with flowing 10 vol.% H_2/N_2 mixed gas at 100 $mL·min^{-1}$ for 1 h. After that the catalysts were denoted as Ce/USY-R, Na-Ce/USY-R, Pd-Ce/USY-R and Pd-Na-Ce/USY-R.

Text S2. Catalyst characterization.

Before characterization, the samples were reduced in 10 vol.% H_2/N_2 mixed gas at 350°C for 1 h.

The surface areas and pore characteristics of catalysts were measured by N_2 adsorption/desorption analysis at -196°C using a physisorption analyzer (BELSORP-max). Before the N_2 physisorption, the catalysts were degassed at 300°C for 1 h. The surface area was calculated using the Brunauer-Emmett-Teller (BET) method.

High-angle annular dark-field scanning transmission electron microscopy (HAADF-STEM) images and element distributions were obtained on JEOL JEM 3200FS TEM with 300 kV acceleration voltage.

X-ray powder diffraction (XRD) patterns of the catalysts were collected with a PANalytical X'Pert PRO X-ray diffractometer (Cu K_α as radiation resource, $\lambda = 0.154$ nm) at 40 kV and 40 mA. The patterns were measured over the 2θ range from 5° to 90° with a scanning step size of 0.02°.

H_2 temperature-programmed reduction (H_2-TPR) was performed in a Micromeritics AutoChem II 2920 apparatus, equipped with a thermal conductivity detector (TCD) and mass spectrometer detector (MS).

For O_2 temperature-programmed desorption (O_2-TPD), the samples were first reduced with 10 vol.% H_2/Ar at 350°C for 1 h, followed by purging with He for 30 min to desorb H_2. The temperature was then cooled down to 25°C, and then the gas was switched to O_2 for adsorption for 60 min. After that, He flowed for 1 h to remove weakly adsorbed O_2. The temperature was increased to 1000°C at a heating rate of 10 °C/min.

Electron spin resonance (ESR) analysis was carried out on a JEOL JES-FA300 ESR spectrometer. The ESR spectra were performed at a microwave frequency of 9.85 GHz at 77 K without light irradiation.

X-ray photoelectron spectra (XPS) were measured by an AXIS Ultra system, equipped with Al Kα radiation (hν = 1486.6 eV) with an X-ray anode operated at 225 W and 15 kV. The C1s peak (284.8 eV) was used to calibrate the binding energy (BE) values.

In the HCHO-TPD experiment, the reduced catalysts (50 mg, 40-60 mesh) were pretreated with gas flow contain 20 vol. % O_2 + 150 ppm HCHO at room temperature

for 1 h. Subsequently, the catalyst was further cooled down to 10°C and purged with He for 30 min. Finally, the HCHO-TPD procedure was performed using He within the temperature range of 10 to 450°C, with a heating rate set at 10 °C/min. A mass spectrometer was employed to monitor the products.
